# Perspective of Future Potent Therapies for Fuchs Endothelial Corneal Dystrophy

**DOI:** 10.2174/1874364101812010154

**Published:** 2018-07-23

**Authors:** Naoki Okumura, Ryousuke Hayashi, Noriko Koizumi

**Affiliations:** Department of Biomedical Engineering, Faculty of Life and Medical Sciences,Doshisha University,Kyotanabe,Japan

**Keywords:** FECD, ECM, Endothelial Corneal Dystrophy, TCF4, SNP, ICD3

## Abstract

**Background:**

Fuchs Endothelial Corneal Dystrophy (FECD) is a progressive disease that affects the corneal endothelium in both eyes. Recent studies have identified a novel genetic basis for FECD, and basic research findings have provided evidence for its underlying pathophysiology. Since its first description by Ernst Fuchs in 1910, the only therapeutic choice has been corneal transplantation using donor corneas. However, accumulating evidence suggests that a change in this “rule” may be imminent.

**Conclusions:**

This article reviews the current knowledge of the genetics and pathophysiology of FECD, and it introduces some potent therapeutic modalities that show promise as new treatments for this disorder.

## 1 INTRODUCTION

Fuchs Endothelial Corneal Dystrophy (FECD) is a progressive disease that affects the corneal endothelium in both eyes. The hallmarks of FECD in the clinical setting are the formation of excrescences, called guttae, on Descemet’s membrane and the loss of corneal endothelial cells. FECD shows a gender dichotomy, with a female to male ratio of 2.5:1 to 3:1. FECD typically occurs at the age of 40–50, and it progresses to an advanced stage in some, but not all, patients. In the advanced stage, decompensation of the corneal epithelium disrupts the water balance in the corneal stroma, which induces edema of the corneal stroma and epithelium and causes severe vision loss [1]. Even in patients without corneal edema, the formation of guttae and the thickening of the Descemet’s membrane due to accumulation of Extracellular Matrix (ECM) components results in forward light scatter and a loss of vision quality [2, 3].

Recent studies have indicated an association between a novel genetic pattern and the development of FECD. In addition, basic research studies are now beginning to reveal the underlying pathophysiology of this disease. Despite these advances in understanding the nature of FECD, the only therapeutic choice for its treatment remains corneal transplantation using donor corneas-the original treatment used when Ernst Fuchs first described this disease in 1910 [1]. However, accumulating evidence now indicates that important changes are imminent regarding the treatment of FECD. This article provides a review of the current knowledge of the genetics and pathophysiology of FECD, and it introduces some potent therapeutic modalities that show promise as new treatments for FECD.

## 2 METHODS

Literature searches were performed in PubMed (https:// www.ncbi.nlm.nih.gov/ pubmed) and ClinicalTrials.gov (https://clinicaltrials.gov/). Key search terms were the references cited in each eligible article that included “Fuchs endothelial corneal dystrophy,” “TCF4,” “single nucleotide polymorphism, SNPs, or polymorphism.” This review is not a meta-analysis; therefore, we selected literature that allowed us to introduce future perspectives as well as to summarize the current status of this research topic.

## 3 GENETICS

Studies of familial FECD cases show that the disease has an autosomal dominant inheritance pattern [4]. However, a certain proportion of patients with FECD have sporadic disease, without a familial history. The ICD3 classification categorizes FECD patients as those with: 1) early-onset FECD, 2) identified genetic loci, and 3) disease without known inheritance [5].

Early-onset FECD is a rare form, and these patients exhibit corneal edema by the age of 30–40. Genetic screening of a family with early-onset FECD identified a missense mutation of the *COL8A2* gene that resulted in substitution of a lysine for a glutamine (Q455K) on chromosome 1 p34.3-p32. Analysis of large families with a common form of late-onset FECD also showed a significant linkage between the *FCD1*, *FCD2*, *FCD3*, and *FCD4* loci on chromosomes 13, 18, 5, and 9, respectively [6-9]. Four genes (*SLCA411*, *TCF8*, *LOXHD1*, and *AGBL1*) were reported as causal genetic mutations, although these genetic mutations were rarely identified in patients with FECD [9-14].

Researchers have since devoted their efforts to identify a genetic cause for the large proportion of patients with FECD. For example, a Genome-Wide Association Study (GWAS), conducted by Baratz and colleagues in 2010, identified a significant association between late-onset FECD and the intronic Single Nucleotide Polymorphism (SNPs) rs613872 in Transcription Factor 4 (*TCF4*) [15]. Similarly, replication studies have shown a strong association between rs613872 and FECD, mainly in Caucasian cohorts [16, 17]. Other SNPs in *TCF4,* apart from rs613872, were associated with FECD in populations from Singapore, Southern China, and India, suggesting the occurrence of ethnic variations in the SNPs in *TCF4* [18-20].

In 2012, Wieben and colleagues reported the discovery of an expanded CTG trinucleotide repeat in intron 3 of *TCF4* in patients with FECD [21]. Their investigation of 66 FECD cases and 63 unaffected controls demonstrated a sensitivity and specificity of 79% and 96%, respectively, for more than 50 repeats identifying FECD; this specificity was higher than that previously reported for the rs613872 SNP. The percentages of patients with FECD that harbored the CTG trinucleotide repeat expansion varied with their ethnicities; however, this strong association was replicated in multiple ethnic groups (Table **1**) [20, 22-27].

## 4 PATHOPHISIOLOGY

### 4.1 The Unfolded Protein Response

Under normal conditions, proteins undergo folding in the lumen of the Endoplasmic Reticulum (ER), and correctly folded proteins are then packaged into ER exit vesicles for delivery to membranes for extracellular secretion. By contrast, improperly folded proteins are removed in the proteasome through the process known as ER-Associated Degradation (ERAD) [28-30]. However, impairment of homeostasis induces ER stress, which disrupts ERAD and triggers apoptosis to remove rogue cells that accumulate unfolded proteins [31, 32]. This highly regulated process is called the Unfolded Protein Response (UPR), and the UPR is involved in the pathogenesis of various diseases, including Alzheimer’s disease, Parkinson’s disease, diabetes mellitus, multiple myeloma, and retinitis pigmentosa [33-38].

In 2010, Engler and colleagues postulated that activation of the UPR plays a central role in the pathogenesis of FECD through the induction of apoptosis in corneal endothelial cells [39]. They showed an increased and dilated ER structure and upregulation of the markers of the UPR in the corneal endothelium of patients with FECD [39]. The same research group subsequently showed that homozygous knock-in of *Col8a2^Q455K/Q455K^*, a causal gene for early-onset FECD, was sufficient to induce FECD-like ocular features in mice, and these changes were linked with UPR-associated apoptosis [40]. Recently, our group showed that ECM components, such as type I collagen and fibronectin, form aggregates of unfolded proteins in the corneal endothelium of FECD patients [41]. We also showed that activation of transforming growth factor-β (TGF-β) signaling causes a chronic overload of ECM proteins within the ER, which induces an accumulation of unfolded protein and activation of the intrinsic apoptotic pathway through the UPR [42]. The genetic background underlying the UPR induction is not yet elucidated; however, accumulating evidence supports a role for the UPR and associated apoptosis in FECD.

### 4.2 Oxidative Stress and Mitochondrial Dysfunction

Much research has confirmed an involvement of oxidative stress in the pathogenesis of FECD, and a linkage between mitochondrial dysfunction and the generation of oxidative stress is suggested [43-47]. For instance, an early study indicated that the numbers of mitochondria were decreased in parallel with downregulation of cytochrome oxidase [48]. Serial analysis of gene expression revealed that expression of mitochondrial antioxidant genes was diminished in the corneal endothelium of patients with FECD [49]. Jurkunas and colleagues demonstrated that an oxidant-antioxidant imbalance leads to oxidative DNA damage and apoptosis [44]. Very recently, Benischke and colleagues reported that constitutive activation of mitophagy causes a reduction in mitochondrial mass and depletion of the numbers of functional mitochondria [50]. Accumulating evidence suggests that oxidative stress, induced by mitochondrial dysfunction, damages corneal endothelial cells. Interestingly, ER stress activates the intrinsic apoptotic pathway (the mitochondrial pathway) in the corneal endothelium, as it does in other cell types [41]. Future studies will likely elucidate the nature of the involvement of both ER stress and oxidative stress in the pathogenesis of FECD.

### 4.3 RNA Foci

In 2015, Du and colleagues reported that the corneal endothelial cells of patients with FECD harbor poly(CUG)_n_RNA, and this results in the formation of RNA foci. They also suggested that RNA toxicity and missplicing play an important role in the pathogenesis of FECD, similar to that played in myotonic dystrophy type 1, a trinucleotide repeat expansion disease [51]. Mootha and colleagues also identified RNA foci in the corneal endothelium of subjects with FECD who showed trinucleotide repeat expansion in TCF4, but these foci were absent from the corneal endothelium of subjects with FECD but without this trinucleotide repeat expansion [52].

## 5 FUTURE TREATMENTS

### 5.1 Current Therapy

Corneal transplantation using donor corneas is currently the only therapy for treating corneal endothelial decompensation diseases, including FECD. Penetrating keratoplasty, in which a full-thickness patient cornea is replaced with full-thickness donor cornea, has been performed since 1906. New surgical procedures, such as Descemet’s Stripping Endothelial Keratoplasty (DSEK) and Descemet’s Membrane Endothelial Keratoplasty (DMEK), selectively replace the diseased corneal endothelial layer with a lamellar donor graft that includes the corneal endothelium. These lamellar surgeries have advantages over penetrating keratoplasty, and their use has therefore spread explosively [53].

### 5.2 Tissue Engineering Therapy

The evolution of corneal transplantation procedures now enables less invasive treatment with better clinical outcomes, but problems remain. The most serious are the shortage of donor corneas, the difficulty of the surgical procedure, and the incidence of graft failure in acute and chronic phases. These issues have motivated researchers to devise tissue engineering treatments that can overcome the current transplantation limitations [53, 54].

Two strategies adopt the use of transplanted cultured corneal endothelial cells as regenerative medicine: 1) transplantation of a cultured corneal endothelial sheet by a procedure resembling DSEK or DMEK and 2) direct injection of cultured corneal endothelial cells, without a carrier, into the anterior chamber [55]. The sheets used in transplantation are produced by culturing corneal endothelial cells on a number of different substrates, such as collagen, amniotic membrane, and human corneal stroma, and animal experiments have confirmed that the transplantation of the resulting sheet enables the regeneration of a transparent cornea [56-59]. However, to the best of our knowledge, the transplantation of cultivated corneal endothelial sheets has not yet been introduced into the clinical setting.

Some research groups, including ours, have attempted to regenerate corneal endothelium directly by injecting cultured corneal endothelial cells into the anterior chamber without a carrier [60-62]. However, animal experiments have revealed that an insufficient number of the injected cells adhere to the back side of the cornea, so that a corneal endothelium fails to regenerate in vivo. Our previous research indicated that cell adhesion is inhibited by the activation of Rho/ROCK signaling, and conversely, inhibition of this signaling pathway by a Rho kinase (ROCK) inhibitor enhances cell adhesion. Therefore, we applied a ROCK inhibitor to promote engraftment [63, 64]. We confirmed, using rabbit and monkey corneal endothelial dysfunction models, that coinjection of cultured corneal endothelial cells and a ROCK inhibitor into anterior chamber resulted in the regeneration of the corneal endothelium [64, 65].

In 2013, after obtaining the approval from the Japanese Ministry of Health, Labour, and Welfare, we initiated a clinical trial (Clinical trial registration: UMIN000012534) at the Kyoto Prefectural University of Medicine to evaluate this cell injection therapy as a treatment for corneal endothelial dysfunction [66]. Our preliminary clinical data have confirmed that coinjection of cultured corneal endothelial cells and a ROCK inhibitor regenerates the corneal endothelium and restores a transparent cornea in human subjects. Further clinical data are necessary, but cell-based therapy appears to be a potent future treatment for corneal endothelial decompensation diseases, including FECD.

### 5.3 ROCK Inhibitor Eye Drops

Rho is a small GTPase, and RhoA activates ROCK, a serine/threonine kinase that phosphorylates various substrates. ROCK signaling plays an essential role in several fundamental cellular events, such as cell adhesion, motility, proliferation, differentiation, and apoptosis [67-69]. In 2009, we reported that inhibition of ROCK signaling promotes the *in vitro* proliferation of corneal endothelial cells [63]. Subsequently, we found that administration of a ROCK inhibitor in eye drop form promotes wound healing in the corneal endothelium in rabbit and monkey models [70-72].

We have since conducted pilot clinical research to investigate the use of topically applied ROCK inhibitor eye drops in patients who have undergone central corneal endothelium removal by transcorneal freezing. Our findings suggested that the eye drop form of ROCK inhibitor is a potent therapeutic treatment choice for patients with early-stage FECD [71, 73]. Notably, one 52-year-old male patient diagnosed with late-onset FECD recovered full corneal transparency after transcorneal freezing and the use of ROCK inhibitor eye drops. His central corneal thickness was reduced from 703 μm to 568 μm and his visual acuity improved from 20/63 to 20/20. His corneal transparency was maintained for more than 6 years, and the original plans for an eventual corneal transplantation were canceled [73].

Recent investigations have examined the effect of surgical removal of the central Descemet’s membrane, including pathological corneal endothelium [74-76], and one clinical study has indicated a positive effect of combining ROCK inhibitor eye drops with this procedure [77]. However, clinical data for the usefulness of ROCK inhibitors in FECD treatment remain limited, so randomized clinical trials are still needed before adoption of this eye drop as a routine therapeutic option.

### 5.4 Potent Pharmaceutical Agents

Stealth BioTherapeutics (Newton, MA) has been developing drug candidates for targeting diseases associated with mitochondrial dysfunction. They initiated a phase 2 clinical trial of elamipretide in eye drop form, with the expectation that this drug would target the inner mitochondrial membrane to help preserve mitochondrial energetics (http://www.stealthbt.com/). The results of this clinical trial have not yet been released, but elamipretide is currently the most advanced-stage pharmaceutical aimed at the treatment of FECD.

Kim and colleagues reported that N-Acetylcysteine (NAC), a thiol-containing antioxidant and radical scavenger, rescued cultured corneal endothelial cells from damage mediated by ER and oxidative stress [78]. They also demonstrated that systemic use of NAC suppressed the progression of FECD in early-onset FECD model mice (*Col8a2^Q455K/Q455K^*), thereby providing an in vivo proof of concept of the use of NAC as a potent therapeutic candidate for treatment of FECD [78]. The same research group also showed that the addition of lithium further increased the survival of cultured corneal endothelial cells when ER and oxidative stresses were triggered [79]. A higher corneal endothelial cell density was also maintained in early-onset FECD model mice given a lithium treatment than in a non-treatment group [79]. The researchers suggested that lithium increases the survival of corneal endothelial cells by an upregulation of autophagy; therefore, lithium may represent a new therapeutic agent for the treatment of FECD [79].

More recently, the same group attempted a drug screening based on the postulated FECD pathophysiology involving ER and oxidative stresses and cell death [80]. They induced ER stress with thapsigargin and oxidative stress with hydrogen peroxide in cultured corneal endothelial cells, and then screened 640 compounds found in the Food and Drug Administration (FDA)-approved drug library. They reported that oxotremorine and mefenamic acid were potential survival factors that overcame stress-related cell death.

Our group has reported that activation of TGF-β signaling activates genes that induce the epithelial-mesenchymal transition (EMT). These include *ZEB1* and *SNAI1,* and their induction results in the accumulation of ECM components [81]. This production of ECM components was more strongly upregulated in cell models established from patients with FECD than in control corneal endothelial cells, following exposure of the cells to TGF-β. We recently reported high expression levels of TGF-β isoforms and TGF-β receptors in the corneal endothelium of patients with FECD, and we proposed that activation of TGF-β signaling induces a chronic overload of ECM components, resulting in apoptosis through the UPR [42]. We also showed that inhibition of TGF-β signaling suppressed this accumulation of ECM components and suppressed UPR-mediated apoptosis in cell models established from patients with FECD. These findings suggest that inhibition of TGF-β might be a potent therapeutic option [42]. A summary of the proposed drug candidates for treating FECD is shown in Table **2**.

## CONCLUSION

Corneal transplantation has been the only therapy available for treating FECD for many years. However, recent advancements in tissue engineering techniques may now provide innovative cell-based therapies. “Missing links” still necessitate further investigations, but new information regarding the genetic background and pathophysiology of FECD is rapidly accumulating. Importantly, the recent findings improve the understanding of FECD, but they also guide further investigations aimed at identifying future therapeutic modalities. Indeed, several drug candidates have recently been reported, although none of these drugs has yet been introduced into the market. Corneal transplantations using donor corneas continue to remain the standard treatment, but we believe that the new therapeutic options, such as cell-based therapy and the use of pharmaceutical agents, will provide less invasive and more effective therapies.

## Figures and Tables

**Table 1:** Summary of previous reports of CTG trinucleotide repeat expansion in TCF4.

-	Wieben ED [21]	Mootha VV [22]	Xing C [23]	Nanda GG [20]	Vasanth S [24]	Nakano M [25]	Foja S [26]	Kuot A [27]
Population	Caucasian	Caucasian	Chinese	Indian	-	Japanese	German	Australian
Cases (FECD)	66	120	57	44	574	47	61	189
Controls	63	100	121	108	354	96	113	183
CTG≥40 or 50 (FECD)	79% (CTG>50)	73% (CTG>40)	44% (CTG>40)	34% (CTG>50)	62% (CTG>40)	26% (CTG>50)	77% (CTG>50)	51% (CTG>40)
CTG≥40 or 50 (Control)	3% (CTG>50)	7% (CTG>40)	2% (CTG>40)	5% (CTG>50)	4% (CTG>40)	0% (CTG>50)	12% (CTG>50)	5% (CTG>40)

**Table 2:** Proposed drug candidates for treating FECD.

Candidate Drugs	Status	Proposed Mechanism	Author or Company
Elamipretide	Phase 2 clinical study	Preserves mitochondrial energetics	Stealth BioTherapeutics
Rho kinase inhibitor	Pilot clinical research	Promotes cell proliferation	Okumura N, Koizumi N [71, 73]
N-acetylcysteine	Animal model	Increases cell survival	Kim EC [78]
Lithium	Animal model	Increases cell survival	Kim EC [79]
Oxotremorine	*in vitro*	Increases cell survival	Kim EC [80]
Mefenamic acid	*in vitro*	Increases cell survival	Kim EC [80]
TGF-β inhibitor	*in vitro*	Suppresses unfolded protein mediated cell death	Okumura N [42]
